# Hydrogel-based milliwell arrays for standardized and scalable retinal organoid cultures

**DOI:** 10.1038/s41598-020-67012-7

**Published:** 2020-06-24

**Authors:** S. Decembrini, S. Hoehnel, N. Brandenberg, Y. Arsenijevic, M. P. Lutolf

**Affiliations:** 1 Department of Ophthalmology, University of Lausanne, Jules-Gonin Eye Hospital, FAA, Unit of Retinal Degeneration & Regeneration, Avenue de France 15, 1004 Lausanne, Switzerland; 2 Laboratory of Stem Cell Bioengineering, Institute of Bioengineering, School of Life Sciences, Ecole Polytechnique Fédérale de Lausanne (EPFL), 1015 Lausanne, Switzerland; 3 Institute of Chemical Sciences and Engineering, School of Basic Sciences, EPFL, 1015 Lausanne, Switzerland; 4 Department of Biomedicine, University Hospital Basel & University Basel, Hebelstr. 20, 4031 Basel, Switzerland; 5 Present Address: SUN bioscience, Bâtiment SE-B, Biopôle, Epalignes, Switzerland

**Keywords:** Assay systems, Stem-cell differentiation, Embryonic stem cells, Biomedical engineering, Gels and hydrogels

## Abstract

The development of improved methods to culture retinal organoids is relevant for the investigation of mechanisms of retinal development under pathophysiological conditions, for screening of neuroprotective compounds, and for providing a cellular source for clinical transplantation. We report a tissue-engineering approach to accelerate and standardize the production of retinal organoids by culturing mouse embryonic stem cells (mESC) in optimal physico-chemical microenvironments. Arrayed round-bottom milliwells composed of biomimetic hydrogels, combined with an optimized medium formulation, promoted the rapid generation of retina-like tissue from mESC aggregates in a highly efficient and stereotypical manner: ∼93% of the aggregates contained retinal organoid structures. 26 day-old retinal organoids were composed of ∼80% of photoreceptors, of which ∼22% are GNAT2-positive cones, an important and rare sensory cell type that is difficult to study in rodent models. The compartmentalization of retinal organoids into predefined locations on a two-dimensional array not only allowed us to derive almost all aggregates into retinal organoids, but also to reliably capture the dynamics of individual organoids, an advantageous requirement for high-throughput experimentation. Our improved retinal organoid culture system should be useful for applications that require scalability and single-organoid traceability.

## Introduction

The mammalian neural retina (NR) is a complex light-sensitive tissue that lines the back of the eyeball. It comprises six neuronal cell types arranged on three different nuclear layers. Many genetic mutations cause dysfunction and death of rod and cone photoreceptors (PR), the two key photosensitive cell types, leading to vision decline and often blindness^1^. Over the past decade, there has been significant progress in the development of novel therapeutic strategies, such as those based on gene and cell therapy, to treat diseases affecting retina function and survival^2^. However, despite these important advances, no therapy exists to robustly protect photoreceptors by targeting the molecular mechanisms of PR loss. Indeed, during cell death, several intracellular pathways are activated^3,4^ and in consequence many inherited retinal dystrophies remain untreatable. Strategies to lock one or several key pathways of cell death need to be developed.

Groundbreaking work by Sasai and colleagues has demonstrated that pluripotent stem cells (PSC) can be stimulated *in vitro* to self-pattern into retinal organoids^5^. These 3D multi-layered NR tissue surrogates recapitulate key hallmarks of *in vivo* retinogenesis, including *(i)* the formation of a continuous neuroepithelium from which optic vesicle (OV)-like structures evaginate, *(ii)* OV distal portion flattening and invagination leading to the formation of optic cup (OC)-like structures, and *(iii)* OC maturation into stratified neural retina containing an outer nuclear layer (ONL) with photoreceptors, an inner nuclear layer (INL) composed by interneurons, Müller glia cells and a ganglion cell layer (GCL). Retinal organoids have thus the potential to accelerate ophthalmology research and drug discovery in an unprecedented way by providing an unlimited source of retinal neurons recapitulating their development *in vitro*^6,7^.

However, despite the promising potential of retinal organoids, it is increasingly clear that they also have shortcomings that limit their wider applicability, an extensive variability in differentiation efficiency leading to low reproducibility being perhaps the most critical issue^8^. Various protocols for the generation of retinal organoids have been reported^9–18^, each of which giving rise to different tissues in terms of size, percentage of different cell types and 3D architecture^19^. The high variability may not be surprising in view of the complicated sequence of tissue manipulation steps that are involved in organoid formation in these protocols. For example, mouse retinal organoid formation is typically started in low-adherent U-bottom 96-well plates (NUNC^TM^), followed by a culture in low adherent 6-well plates or 35 mm dishes (day 14–25)^5^ with or without cutting the nascent retinal tissue. The necessary transfer of organoids from one culture vessel to another^5,9^, requiring tedious manual handling steps, renders the standardization or automation of this process challenging. Moreover, it has been reported that during their maturation phase, retinal organoids floating in the same compartment tend to fuse to one another or stick to the culture plate, making differentiation efficiency difficult to coordinate and to thus reliably evaluate different biological actions^9,16,19^.

To address these limitations, here we report a tissue-engineering strategy for the robust and scalable generation of mouse retinal organoids. We introduce topographically structured hydrogel substrates to efficiently aggregate defined numbers of mESC into clusters which, without any additional manipulation step (e.g. change of tissue culture plate), develop into retinal organoid arrays (ROAs). The spatial confinement of organoids in predefined milliwells allows an experimenter to reliably trace the development of retinal organoids with unprecedented accuracy and throughput.

## Results

### Design and fabrication of hydrogel substrates

To design a suitable substrate for the generation of mouse retinal organoids in one step, *i.e*. without any extra manipulation of the tissues as performed conventionally, three critical criteria were identified: *(i)* the capacity to ensure the formation of mESC aggregates from approximately 3000 cells and their subsequent specification into neuroepithelia; *(ii)* the ability to reproducibly induce OVs from these neuroepithelial tissues; *(iii)* the possibility to further mature all the OV tissues over several weeks in the same environment without manipulations but regular medium changes.

**Figure 1:**
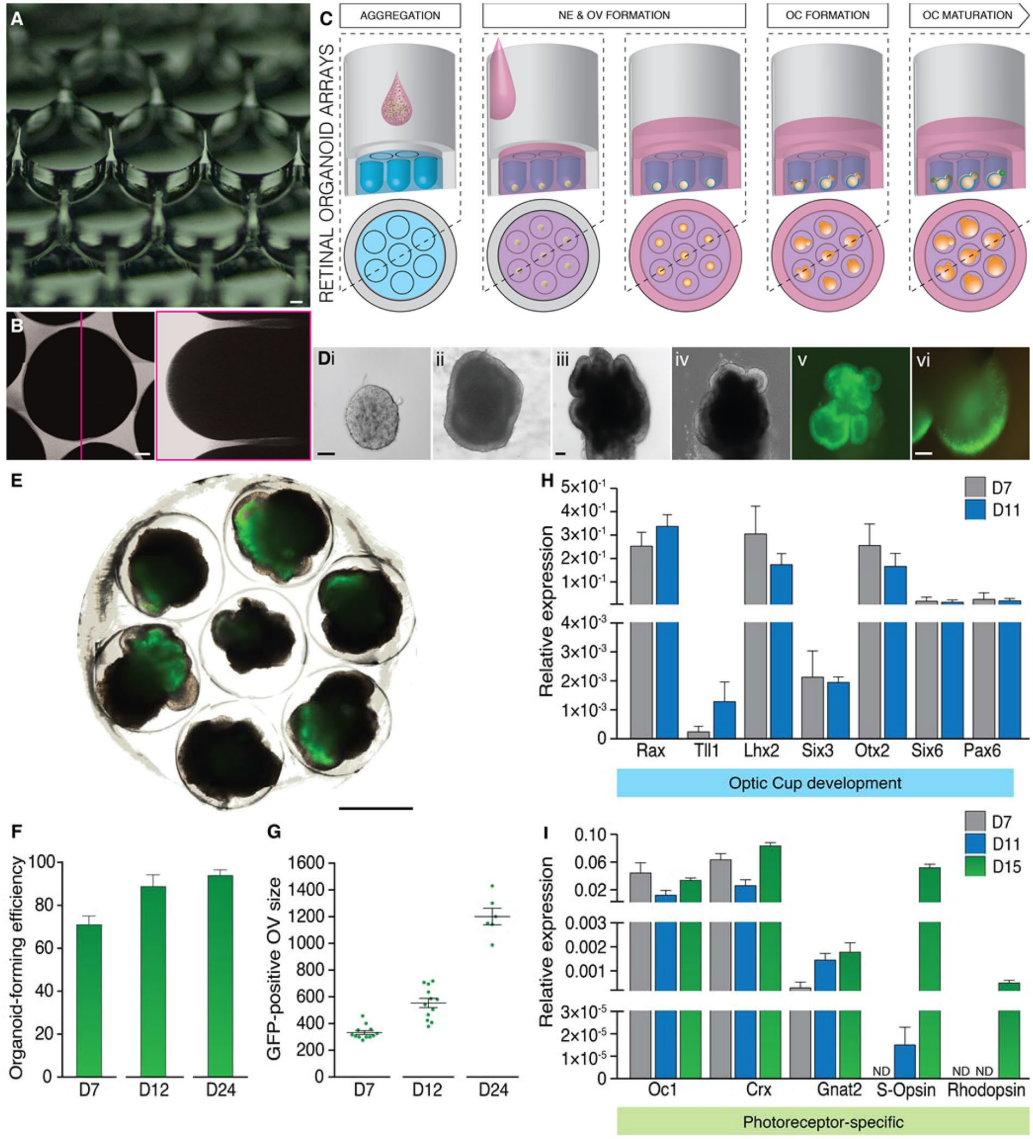
(**A**) Macroscopic photograph of an array of milli-wells in PEG hydrogel. Scale bar: 200 μm. (**B**) Orthogonal reconstruction of a confocal z-stack of a single milli-well to verify their geometrical integrity. Scale bar: 200 μm. (**C**) Schematic representation of our novel approach. (**D**) Time course of Crx-GFP mESC-derived retinal organoids in microwell arrays after protocol optimization. (i) Micrograph of a single aggregate 20 h after seeding. (ii) Formation of a rigid, bright neuroepithelium surrounding the aggregate at day 3 of culture. (iii) Day 4, optic vesicle (OV) protrusion from specialized area of the neuroepithelium. (iv) Day 6, rare optic cup-like (OC) formation. (v) Day 7, first detection of photoreceptor differentiation revealed by Crx-GFP expression. (vi) Developing retina at 26 days of culture showing GFP-positive photoreceptors. Scale bars: 100 μm. (**E**) Tile scan of a retinal organoid milli-well arrays at day 14. Scale bar: 1 mm. (**F**) Quantification of the organoid-forming efficiency, based on the GFP expression, at different days of culture. In D24, ≈93% of the aggregates contained at least one retinal organoid (**G**) Size quantification of the GFP-positive optic vesicles (OV) at different differentiation stages. (**H, I**) Gene expression profiles of ROA-organoids for eye field transcription factors and for cone and rod photoreceptor cells at different differentiation times, i.e. day 7 (D7) in grey, day 11 (D11) in blue and day 14 (D14) in green.

To build a culture system that could accommodate these key steps in organoid development, we used standard microfabrication strategies to generate 1.5 millimeter diameter round-bottom cavities, or milliwells, into hydrogel substrates (Fig. 1A,B). Based on geometrical calculations to maximize cell capture (Fig. S1), we chose a small distance between milliwells and placed seven milliwells within one culture vessel (here: 24-well plates) to increase the density of organoids per plate and thus throughput for future organoid assays. After testing several naturally derived hydrogels to mold milliwell arrays (data not shown), we opted for poly(ethylene glycol) (PEG)-based hydrogels as substrate material due to its intrinsic inertness and modularity in physicochemical characteristics^20^. Indeed, PEG hydrogels could be reliably molded at variable stiffness, without any geometrical aberration.

### Generation of retinal organoids in hydrogel milliwell arrays

We next attempted to generate retinal organoids on our hydrogel-based milliwell arrays to compare their development to organoids reported in the literature^12^. We chose transgenic mESCs expressing GFP under the control of the *Crx* promoter (*Crx*-GFP^16^), a gene that is specifically expressed in developing and post-mitotic photoreceptors, allowing us to track photoreceptor development in live organoids.

We observed that ESCs seeded on milliwell arrays rapidly aggregated to form cell colonies (Fig. 1D and Fig. S2Ai, S2Aii at D0), most likely because of the cell-repellent characteristics of PEG hydrogels^20^. However, from day 2 onwards, cell aggregates remained small, even undergoing slow death (Fig. S2Ai at D5). We hypothesized that lack of cell colony growth and organoid development could be due to a lack of critical survival and growth factors in the densely packed organoid arrays. To test this, we used COMSOL Multiphysics^®^ to model mass transport in the milliwell culture system. This simulation predicted a local shortage of diffusive factors in close proximity of cellular aggregates located at the bottom of milliwells after four days of culture (Fig. S2B). However, we found that a combination of N2 and B27 could overcome the shortage of nutrient and growth factors, allowing for the expected development of retinal organoids emerging from the aggregates (Fig. 1D,E).

### Milliwell arrays to reliably induce and trace single retinal organoids

The above-described milliwell array format was thus selected to simultaneously induce the formation of seven aggregates per macrowell of a 24-well plate. 21 × 10^3^ mESCs seeded in each macrowell reproducibly gave rise to an array of seven aggregates in less than eight hours (Fig. S2Ai and S2Aii, at D0). The addition of basement-membrane matrix components 18 hours after seeding led to the specification of an optically brighter neuroepithelial layer surrounding the aggregate at day 3 of culture (Fig. 1Dii). At day 4, a specialized area of the neuroepithelium evaginated to give rise to OV-like structures (Fig. 1Diii) that rarely flattened and distally invaginated to generate optic cups (OC) (Fig. 1Div). One aggregate can contain one or several OV/OC-like structures surrounded by non-retinal tissue. Interestingly, we observed a high number of GFP-positive eye cup formation already at day 7 (Fig. 1Dv,F), a developmental stage that is reached *in vivo* within 10 days. The growth kinetics of the retinal organoids in milliwells revealed a continued growth all along the entire culture period of 26 days (Fig. 1Di-vi,G).

We next analyzed the developmental stage and maturation of retinal organoids^12,16^ generated on our ROAs. Specifically, we used qPCR analysis to compare expression profiles of key eye field transcription factors involved in the specification of the vertebrate retina^21^ and of cone and rod photoreceptor markers^22^ (Fig. 1H,I). It was previously reported that mouse *Six3* RNA-injected fish embryos develop ectopic retinal tissues^23^ and that expansion of the *Rax* expression domain indicates a retinal identity^24,25^ though *Rax* is also expressed in the ventral diencephalon. At day 7, *Rax* expression was found to be strikingly high, indicative of the relatively rapid OV induction, growth and photoreceptor marker appearance, in line with the marked expression of endogenous *Crx* (Fig. 1I) as early as day 7 (Fig. 1Dv), five days before its expression *in vivo*^5,16,26^. Notably, *Otx2*, a target gene of RAX, was also well expressed at this organoid stage. After 11 days of differentiation, *Rax* expression was still high and *Six3* level increased, in line with the continuous growth of optic vesicles and OC at the same time point (Fig. 1H).

To determine the photoreceptor cell sub-type involved in the precocious differentiation program, we next analyzed *Onecut1* (*OC1*)^27^, a gene implicated in the fate specification of cones (as well as horizontal cells), and *Rhodopsin*, expressed in maturing and mature rods. Interestingly, *OC1* was well present by day 7. In addition, by day 11, the transcription of *Gnat2*, a protein involved in the visual transduction machinery of cone photoreceptors, was already transcribed in organoids grown on hydrogel substrates (Fig. 1I). *S-Opsin*^22^, a protein that confers blue sensitivity to cones, was detected at day 15, along with *Rhodopsin* (Fig. 1I). This sequence of cone and then rod markers mimics the genesis of photoreceptor subtypes found *in vivo*, demonstrating the potential of our system to recapitulate and marginally accelerate the development of photoreceptor-containing organoids.

### Immunohistological analysis of organoid-derived retina layers

**Figure 2:**
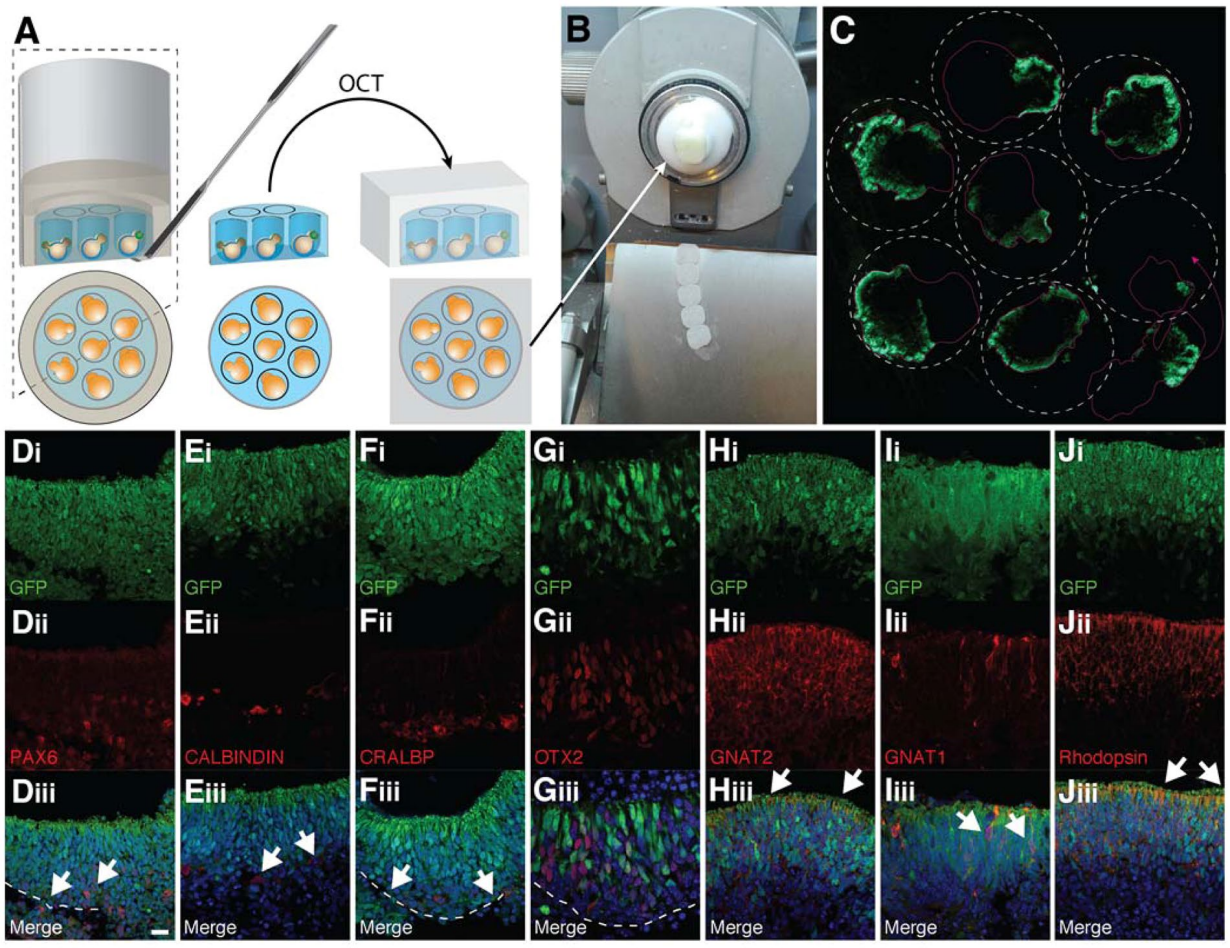
(**A**) Schematic representation and photograph of histological sectioning of ROAs. The entire gel array containing the organoids is embedded in OCT and, (**B**), frozen in order to be cryo-sectioned. (**C**) Representative wide-field fluorescence tile scan of an entire section of a ROA. Green fluorescence represents the GFP expressed in photoreceptor cells. The dotted line represents the milliwell edges. (**D–J**) Immunocytochemistry of *Crx*-GFP-derived retinal organoids cultured on milliwell arrays to detect the retinal cell type composition at day 26 of differentiation. (**Di–Ji**) GFP-positive cells represent the photoreceptors. (**Dii**) PAX6-positive ganglion and amacrine cells, (**Eii**) CALBINDING-positve horizonatl cells, (**Fii**) CRALBP-positive Muller glia, (**Gii**) OTX2-positive, CRX-negative bipolar cells, (**Hii**) GNAT2-positive cone cells, (**Iii**) GNAT1-positive rod photoreceptors in red and, (**Jii**), Rhodopsin-positive photoreceptors in red, their respective merged counterparts (**Diii–Jiii**). White dashed lines indicate the partially occurred lamination process to separate the neuroblastic layer, where GFP-positive photoreceptor localised, from the underneath ganglion cell layer. Scale bar: 100 μm.

Milliwell arrays are potentially ideal substrates to streamline immunohistochemistry on cryo-sections because organoids of each array are located on the same sectioning plane (Fig. 2). This should allow an experimenter to simultaneously cut multiple organoids in parallel, to reduce slide to slide variations and tissue disruption, and to substantially speed up the process. To demonstrate this and characterize the cell type composition in 26-day old organoids, ROAs were cryosectioned and analyzed by IHC-Fr (Fig. 2A–C). Notably, the different cell type percentages were determined exclusively within retina-like tissues (*i.e*. not the whole aggregate) which were identified based on the retinal ganglion cell layer and the outer nuclear layer. In particular, we chose PAX6 as a pan-ganglion and -amacrine cell marker that appears when retinal neurons are formed^28,29^. Interestingly, we found that 14.5 ± 0.7% of PAX6-positive cells were present in the retinal-like tissues (Fig. 2D, n = 22 organoids derived from 4 experiments), consistent with the previous gene expression analysis and literature^16^. Other types of interneurons and glial cells localized within the inner nuclear layer such as CALBINDIN-positive cells (horizontal cells; 1.3 ± 0.4%), CRALBP-positive (Müller cells; (4.5 ± 1.2%) and OTX2-positive/*Crx*-GFP-negative (bipolar cells; 9.6 ± 3.1%) cells, were detected by immunostaining at day 26 (Fig. 2E–G, n = 22 organoids derived from 4 experiments). 80 ± 2% of the cells in the neural retinal region of the organoid were *Crx*-positive photoreceptor cells. Interestingly, this percentage exceeds the one found in mature mouse retinas^26^. Perhaps more importantly, we observed a significant fraction of cells positive for the phototransduction protein GNAT2 (21.5 ± 3.1% of the *Crx*-GFP-positive cells) marking cones (Fig. 2H**)**. Concerning rods, 68.6 ± 3.7% RHODOPSIN-positive and a few GNAT1-positive rod photoreceptors were detected among the photoreceptor-like population in 26 day-old organoids (Fig. 2I,J). Such panel of protein expression is in line with the one observed in PN 4–5 mouse retinas in which a bulk of cones translate GNAT2 and few rods start to express GNAT1^30^.

A further structural element that suggests that *in vitro*-generated retinal tissues at day 26 show similarities with *in vivo* tissues is the retina lamination process^31^. *In vivo*, the separation between the outer and inner nuclear layer is visible after PN6, while the one between the neuroblastic and ganglion cell layer is already present at PN4. Similarly, presumptive age-matched retina-like tissues show a retina structure comparable to PN4 retina (Fig. 2Diii-Jiii dashed lines; 45%-positive for separation). In this culture condition, similarly to our previously reported protocols^16^, the retinal pigmented epithelium (RPE) differentiated from day 11 onward but the structures analyzed showed variations in the extent of RPE development. Notably, after 26 days of culture, almost all organoids show one or more RPE patches (Fig. S3).

**Figure 3:**
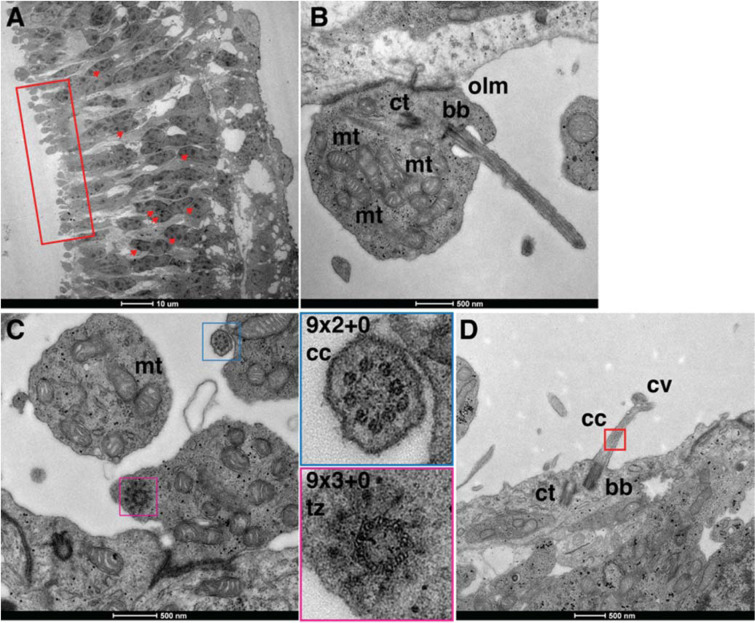
Electron micrographs of *Crx*-GFP-derived retinas at 25 days. 50 nm ultra-thin sections were imaged by standard EM section of resin embedded retinal organoids. (**A**) Retinal-like section exhibits characteristic cone morphologies with several polymorphic heterochromatin foci (representative examples shown with arrowheads). The insert highlights cone inner segments. Scale bar: 10 μm. (**B**) A PR inner segment with mitochondria (mt), a centriole (ct), a basal body (bb) and outer limiting membrane (olm). (**C**) Classical array of nine triplet and doublet microtubles of bb and transiction zone (tz) of the cc respectively. (**D**) A PR with a connecting cilium (cc) bearing a rudimentary outer segment, the ciliary vesicle (cv). Scale bars (**B,C**) = 500 nm.

Finally, 50-nm sections were used to study the outer retina ultrastructure by electron microscopy. Retinal organoids at day 25 showed thick external layers tightly packed with cells, showing several polymorphic heterochromatin foci typical of cone-like photoreceptors (Fig. 3A; red arrows). Rare rod-like photoreceptor morphology with highly condensed heterochromatin was detected (data not shown). Inner-segment structures with high density of mitochondria were observed (Fig. 3A; insert). Below the inner segment, electron-dense structures, corresponding to the outer limiting membrane (OLM) formed by footplates of Müller glia cells, were detected (Fig. 3B). The photoreceptors show connecting cilia (cc) with the characteristic ‘9 + 2' and “9 + 3” microtubule arrays of the basal bodies (bb) and of the transiction zone (tz), respectively^32^ (Fig. 3C-C’-C”). At the tip of the connecting cilium, we often detected the presence of a rudimentary outer segment, the ciliary vesicle (Fig. 3D), which, although still somewhat immature, are reminiscent of disc formation during mid-stages of mouse OS development^33^. As expected, due to the immature stage of the retinal organoids at day 25, no ribbon synapses were detected.

## Discussion

In this study, we present a novel hydrogel-based platform to generate reproducible and scalable mouse retinal organoid cultures, termed ROAs. Existing methods to generate mouse or human PSC-derived organoids are costly, manual labor-intensive and thus nearly impossible to scale notably using robotized systems. In the present work, we aimed at developing a versatile platform technology to address these shortcomings. By using standard microfabrication processes, we could reliably emboss millimeter-sized, round-bottom cavities into hydrogel substrates. A small inter-milliwell spacing ensured a highly efficient cell trapping which we think is crucial for controlling the starting cell number involved in organoid morphogenesis. Moreover, having multiple, evenly spaced organoids within the same culture compartment increased the number of organoids generated per unit area and allowed for the reliable tracing of individual organoids during their entire development (3–4 weeks).

Our technology offers key advantages over currently available methods for PSC-derived organoid culture. While conventional methods rely on standard plastic plates and frequent transfer of organoids into different cell culture vessel^5,34^, previously developed microwell technologies are designed for creating cell aggregates^35,36^ but cannot accommodate for the tremendous growth that PSC-derived organoids undergo and do not support tissue morphogenesis within the same culture vessel^37^. The significant benefit of this technology is the requirement of an easy-one-step handling to generate retina organoids without successive intervention. This approach will allow automation of the entire culture process from cell seeding to regular medium changes as well as the monitoring of retinal organoid growth and differentiation.

Notably, organoids cultured individually grew regularly, developed a retina structure with all the retinal cell types and appeared to respect the chronological birth of photoreceptor subtypes. The physicochemical characteristics of the PEG hydrogels used in this protocol allowed for a good diffusion of metabolites as well as factors which may favor organoid development. Moreover, comparing the appearance of retinal structure and cell types with published protocols, induction of OVs and the generation of *Crx*-GFP-positive cells seems to appear rapidly (after only 4 and 7 days, respectively). In addition, the percentage of cones, which are essential for day and color vision and high visual acuity, is elevated, reaching around 20% of the Crx-GFP-positive cells. This provides for a unique opportunity to study cones which are of low abundance in the mouse retina (3% of the photoreceptor population).

The reasons for the relatively high number of cones and their early maturation in our setting might be related to the use of B27 without vitamin A. Indeed, B27 contains 21 compounds, including important survival factors and photoreceptor related compounds such as Insulin and Vitamine E and T3^38,39^, which is required for cone induction. In mammals, S-cones are the first photoreceptor subtype to be specified. The presence of T3 may be partially responsible for the early cone maturation and their high abundance in our culture in general. Indeed, it has recently been demonstrated that rod photoreceptors in mice are derived from the S-cone precursor lineage via the transcription factor NRL to augment rod photoreceptor number^30^. T3 may have diverted these S-cone precursors towards a cone fate instead of rods.

The observed acceleration of cone maturation compared to our previous study^16^ may be particularly relevant for human retinal organoid development. Indeed, human photoreceptor development *in vitro* is overly long. Thus, the availability of a protocol to achieve a quicker differentiation of human cones and rods, together with the high degree of scalability and automation reached with the presented setting in ROAs, may provide exciting perspectives for the study of cone development and disease modeling.

In the current format, we provide 7 milliwells in one ROA per macrowell of a 24-well plate. The same ROA format can be adjusted to a 96-well plate format for a throughput that is amenable to drug screenings, as recently demonstrated with patient-derived colorectal cancer organoids^40^. We believe that the presented platform will enable the robust screening of morphogens and other compounds that may speed up differentiation and maturation of cell types of hPSC-derived retinal tissues.

## Materials and Methods

### Preparation of hydrogels

PEG hydrogels crosslinked via Michael-type addition reaction were prepared as described^20^, mixing aqueous solutions containing thiol- and vinylsulfone- functionalized 4-arm and 8-arm PEG macromers (mol. weight 10 kDa) at various concentrations to adjust stiffness and stoichiometric ratio. Here, a v/w ratio of 7.5%, corresponding to an average shear modulus of G′ ≈ 20kPa, was used to fabricate the milliwell arrays. The solution was deposited and molded as explained above. The construct was crosslinked for 15 minutes at room temperature.

### Fabrication of U-shaped hydrogel milliwell arrays

U-shaped hydrogel milliwell arrays were fabricated as described elsewhere^40^. Briefly, U-shaped cavities of 1.5 mm in diameter, 1.8 mm in depth spaced by 75μm were generated onto standard 4-inch silicon wafers using standard Si Bosch and soft lithography processes. PDMS (ratio 1:10) was poured onto the wafers and cured overnight at 75 °C. After crosslinking, the PDMS stamps were de-moulded and punched with various diameters. The desired stamps were mounted on custom-made holders. The final non-crosslinked hydrogel mix was deposited onto the PDMS stamp, and the holder-stamp-hydrogel construct was inserted into a custom-made PDMS ring, placed at the bottom of wells of a 24 well plate. The hydrogels were incubated at 37 °C and 5% CO_2_ for 15 minutes (see below). After crosslinking, aqueous buffer (e.g. 1X PBS) was pipetted into the wells and the stamps were removed carefully. The resulting microwell arrays were sterilized thoroughly in buffer under UV light and stored at 4 °C until use.

### ESC culture

ESC-Crx-GFP^16^ were split twice on MEF and twice on gelatin before aggregate formation on milli-arrays or expanded onto MEF prior to cryo-preservation to be stored. Briefly: 6 well plates (Sigma, CLS3516 SIGMA Corning® Costar®) were coated with 0.1% of Gelatin (Sigma, G2500) for 30 minutes at 37 °C and aspirated. Fibroblasts were plated in MEF medium until confluence and inactivated 2 h at 37 °C with Mitomycin C (Sigma, M0503). The day after inactivation, ESCs were plated on inactivated feeder layer in ESC maintenance medium. After two days, when the cells reached 80% confluence, the medium was changed, cells washed in phosphate-buffered saline (PBS, Gibco, 10010-023) and trypsinized (Gibco, 25200-056). Re-suspended cells were placed in a fresh ESC maintenance medium to achieve a split ratio of 1:10 onto plates with a new feeder layer or 1:6 on gelatin. ESC maintenance medium was composed as follows: 500 ml GMEM (Gibco, 11710-035), 5.1 ml 100X non-essential amino acids (NEAA, Gibco, 11140-050) , 5.1 ml 100mM  sodium pyruvate (Gibco, 11360-039), 0.51 ml of 0.1 M beta-mercaptoethanol (2-ME, Gibco, 31350-010), 58 ml knockout serum (KSR) and 5.8 ml FCS were sterilized by filtration through a 0.2 μm bottle-top filter (Millipore Corporation), stored at 4 °C and used within 1 month^5,12^. 2.000 U/ml and 4.000 U/ml of LIF was freshly added to the culture medium each time at medium change on MEF and on gelatin, respectively.

### Retina organoid media

#### Optic vesicle (OV) induction medium^16^

From day 0 to days 7, ESCs were kept in a medium composed of 500 ml GMEM, 5.1 ml non-essential amino acids, 5.1 ml sodium pyruvate, 0.51 ml of 0.1 M 2-ME, N2 (1%, Thermo fisher, 17502-048), B27 minus vitamin A (1%, Thermo fisher, 12587001) and 7.6 ml KSR.

#### Optic cup (OC) induction medium^16^

From day 7 to day 9 the aggregates were incubated in DMEM/F-12 with GlutaMAX (Gibco, 10565-018), and 1% of N2 supplement (Gibco, 17502-048).

#### Retinal maturation medium

From day 9 to day 12, aggregates were kept in DMEM/F-12 with GlutaMAX (Gibco, 10565-018), and 1% of N2, 2% B27 supplement (Gibco, 17502-044). From day 12 onwards, further maturation of the optic cups was stimulated by adding fresh Taurine (Sigma, T8691, 1 mM) to the retinal maturation medium and removing the B27 supplement. Further, from day 12 aggregates were kept in hyperoxic conditions (40% of O2, Sanyo, cat. no. MOC-175M). The optic cup morphogenesis and the following maturation were allowed to proceed inside the mother aggregate until day 24-26, when optic cups where analyzed.

### Procedures established for ROAs

#### Milli-well plates equilibration

Milli-well plates, composed of 7 micro-cavities, were quickly washed twice in PBS and equilibrated in 140 μl of OV induction medium without supplements (see below) at 37 °C for 30 minutes.

#### Retinal organoid protocol in milliwell arrays

In the meanwhile, *Crx-*GFP mESCs^16^ were washed with phosphate-buffered saline (1X PBS, Gibco) and detached with trypsin (Gibco, Cat. no. 25200-056). The cells were then resuspended in Optic Vesicle (OV) induction medium at a density of 525′000 cells/mL in order to seed 3000 cells per microwell. 40 μl of the cell suspension was then added on top of the arrays, in the seeding compartment, and the cells were left to sediment for 30 minutes at 37 °C and 5% CO_2_. Then, 660 μL of OV induction medium was added. Aggregates form as soon as 8 h after seeding. We adjusted the amount of medium needed per well to 840 μL to match the amounts of media per organoids reported in the literature during the first 10 to 14 days of differentiation in 96 U-bottom plates (*i.e*. 120 μL). For this reason, after an overnight incubation, the cells formed aggregates in each microwell and 140 μL of a diluted growth factor reduced Matrigel^TM^ solution (12%, Corning) was added in each of the 24 wells, where the final Matrigel concentration was 2%. The aggregated cells were left in OV induction medium for 7 days. At day 7, the medium was changed to OC induction medium and left until day 12. At day 12, the medium was subsequently changed to Retina Maturation medium as previously described^16^, until day 24-26. In this case, the medium was changed every other day. Additionally, the organoids were incubated in hyperoxic incubators from day 12 on, to promote the survival of newly born photoreceptors^5,16^.

### Individual retinal organoids, whole array fixation and immunohisto/cytochemistry

Individual retinal organoids and whole arrays were fixed with 4% paraformaldehyde (PFA) in PBS for 15 minutes at room temperature (RT) and cryoprotected by successive incubation in 10%, 20%, and 30% sucrose in PBS at least overnight at 4 °C and then embedded in yasulla (30% egg albumin and 3% gelatin in distilled water water) and snap-frozen in dry ice before sectioning. 12 μm cryosections were cut in a cryostat (Thermo Scientific Microm). The sections were stored at −80 °C until use.

12 µm sections prepared on Superfrost plus glass slides were incubated for 1 h in blocking buffer (0,1–0,3% Triton X-100, 1–10% goat, rabbit, or sheep serum, and 0.1–0.5 mg/mL of bovine serum albumin (BSA) diluted in PBS), and incubated ON at 4 °C or RT or 3 hours at RT with primary antibodies (suppl. Table 1). Sections of retina were used for immunohistochemical analysis to confirm antibody specificity. The following antibodies were used: PAX6 (Covance, rabbit, 1:300), RHODOPSIN (Santa Cruz, mouse, 1:300), OTX2 (Abcam, rabbit, 1:300), GNAT2 (Santa Cruz, rabbit, 1:200), GNAT1 (Santa Cruz, rabbit, 1:1000), CALBINDIN D28k (Labome, rabbit, 1:500), CRALBP (Abcam, mouse, 1:500). The nuclei were counterstained with 4',6-diamidino-2-phenylindole (DAPI). Control sections were incubated with secondary antibodies only. Stainings were observed and pictures acquired using a confocal (LSM700, Zeiss).

### Microscopy, image processing and quantification

Images were acquired with a LSM700 invert laser scanning confocal (Zeiss). Confocal microscopy was performed with similar settings for laser power, photomultiplier gain and offset, with a pinhole diameter of one Airy unit. Thin optical sections were used for channel co-localization. Images were adjusted for brightness and contrast using ImageJ (NIH; http://rsb.info.nih.gov/ij/).

### RNA isolation and reverse transcription

Seven organoids collected from one ROAs and from 96 well plates at different day of differentiation, respectively day 7, 11, 15, were analysed in triplicates. RNA was extracted from the retinal organoids according to the manufacturer’s instructions using TRIzol reagent (Invitrogen-Thermofisher, Zug, Switzerland, 15596-026). RNA concentration and OD260/280 ratio were determined using a Nanodrop 1000 (Thermo-Scientific).

During reverse transcription, to denature any secondary structures in the RNA template, 1 µg of total RNA, and 1 µl of random hexamer primers (Promega, Madison, USA; 0.5 mg/ml) were incubated at 70 °C for 10 min and immediately cooled on ice. For the reverse transcription reaction, 4 µl AMV-RT buffer (Promega, Madison, USA), 2 µl dNTP mix (Promega, Madison, USA; 10 mM), 1 µl RNAse Inhibitor (RNAsin; Promega, Madison, USA), and 1 µl AMV reverse transcriptase (AMV-RT; Promega, Madison, USA) were added to a 20 µl final volume reaction and incubated at 42 °C for 1 h, followed by 10 min incubation at 95 °C.

### Quantitative real-time RT-PCR (qPCR) analysis

Primers were designed using the Primer 3 (Simgene.com) software, spanning an exon–exon junction where applicable (see Supplementary Table 1 for mouse qPCR primer list). Primers were synthesized by Microsynth (Microsynth AG, Balgach, Switzerland). For qPCR, we used a 1:30 dilution of the synthesized cDNA.

qPCR was carried out using LightCycler® 480 SYBR Green I Master (Roche, Zug, Switzerland, 04887352001) with the following protocol: 95 °C 600 s; 45 × : 95 °C 10 s, 60 °C 10 s, 72 °C 10 s; 95 °C 10 s, 65 °C 60 s, 97 °C 1 s, 37 °C 30 s. The reaction mixtures were set up according to the manufacturer’s recommendations (Roche, LightCycler® 480, Zug, Switzerland). Primers were synthesized by Microsynth (Microsynth AG, Balgach, Switzerland). Amplification products were resolved on a 1.5% agarose gel to check the band. All PCR experiments were performed at least 3 times on at least 3 independent cell samples. All expression values were normalized to the value of Erl8 gene. Relative normalized expression Basic ∆∆C -Method and the standard error of mean (s.e.m) were calculated using Roche integrated software.

### Electron microscopy (EM)

Retinal organoids were incised using a microsurgical knife (Oasis Medical, Inc. cat no. PE3015) before fixation in 2.5% glutaraldehyde – 2% paraformaldehyde in 1X PBS at 4 °C overnight. The organoids were then washed three times in Cacodylate buffer (0.1 M, pH 7.4) for five minutes and post-fixed in 1% osmium tetroxide and 1.5% potassium ferrocyanide in Cacodylate buffer (0.1 M, pH 7.4) at room temperature (RT) for 40 minutes. The solution was then changed to 1% osmium tetroxide in Cacodylate buffer (0.1 M, pH 7.4) and the samples were further incubated for an additional 40 minutes at RT. Then, the organoids were washed two times in distilled water for five minutes, stained with 1% uranyl acetate (in H_2_O) and washed again two times in distilled water for five minutes. The samples were then dehydrated in graded alcohol series of 15 minutes each with the following alcohol contents: 50%, 70%, 96% and 100%. Finally, we embedded each sample separately in Durcupan by rotating their respective recipients continuously and adding increasing amounts of resin to the sample initially in 100% EtOH. After rotation, the samples were incubated overnight in a ratio of 1:1 of Durcupan 100% to EtOH 100%. The next day each organoid was incubated in 100% Durcupan for the entire day. The samples were then sectioned, embedded on coated glass slides and incubated in the over at 65 °C overnight. Serial sections were performed and the sections were imaged using a transmission electron microscope (FEI Tecnai Spirit, 120 kV).

### Computational characterization of diffusion on the ROAs

Using COMSOL (COMSOL Multiphysics®), two computational reaction-diffusion models were designed to mimic seven spheres, having the same negative reaction rate, on a hydrogel microwell substrate, characterized by a slower diffusion coefficient than the medium (10X slower), into 840 μL of medium. We simplified the system by assuming that the medium is a single species and that its maximum concentration is normalized. In addition, the organoids, represented as spheres of a constant size, were considered to have a constant medium consumption rate over the time span of interest. We chose to simplify the model as much as possible to only analyze the effect of geometry on the local depletion of a normalized species.

### Statistics and counting

Statistical analyses are based on at least four biological replications and three technical replicates. At least 84 organoids were analyzed per experiment. The percentage of cell type counted in the organoids was related to the retina-like structure and not to the whole aggregate/organoid. For one retinal organoid slice, the retinal ganglion cell layer and the outer nuclear layer served to delineate the area to count. All means are presented with ± SEM (standard error of the mean). Statistical significance was assessed using Graphpad Prism 5 software, and applying one-way ANOVA with Tukey’s correction. In figures, *P < 0.05, **P < 0.01, and ***P < 0.001.

## Supplementary information

Supplementary Information.
