# Supplementary material for: Hydrogel-based milliwell arrays for standardized and scalable retinal organoid cultures
Source: Sci Rep. 2020 Jun 24;10:10275. doi: 10.1038/s41598-020-67012-7 (PMC7314858; doi:10.1038/s41598-020-67012-7)
Supplement: Supplementary file 1 — Supplementary Information. [file 41598_2020_67012_MOESM1_ESM.pdf]

Supplementary Information for

Hydrogel-based milliwell arrays for standardized and scalable retinal organoid cultures

Decembrini S[1,4,5], Hoehnel S[2,5], Brandenberg N[2,5], Arsenijevic Y[1,*], Lutolf MP[2,3,*]

[1]Department of Ophthalmology, University of Lausanne, Jules-Gonin Eye Hospital, FAA, Unit of Gene Therapy & Stem Cell Biology, Avenue de France 15, 1004 Lausanne, Switzerland

[2]Laboratory of Stem Cell Bioengineering, Institute of Bioengineering, School of Life Sciences, Ecole Polytechnique Fédérale de Lausanne (EPFL), 1015 Lausanne, Switzerland.

[3]Institute of Chemical Sciences and Engineering, School of Basic Sciences, EPFL, 1015 Lausanne, Switzerland.

[4]Department of Biomedicine, University Hospital Basel & University Basel, Hebelstr. 20, 4031, Basel, Switzerland.

[5]The authors contributed equally to the presented work

*To whom correspondence should be addressed: matthias.lutolf@epfl.ch or yvan.arsenijevic@fa2.ch (for retinal organoids)

Supplementary Figures

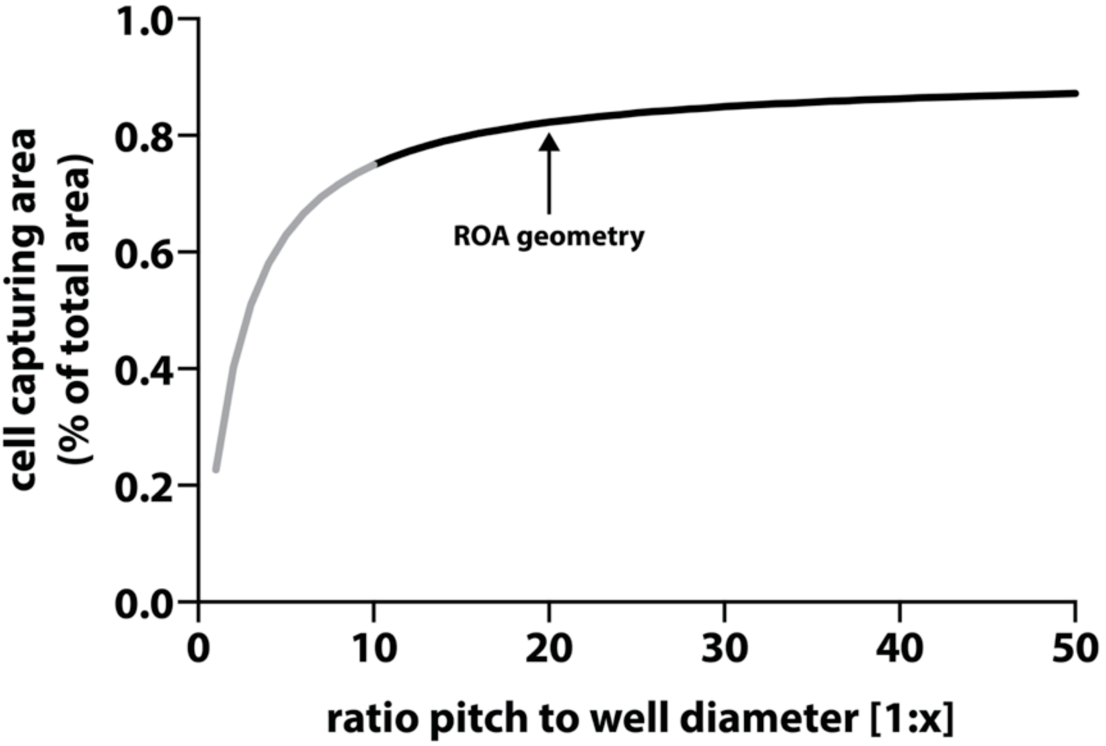

Supplementary Figure 1: Geometrical relationship between the {pitch or inter-well distance}:{well diameter} ratio (i.e. {well diameter} divided by the {pitch}) and the percentage of the array surface that contains milliwells, named “cell capturing area”.

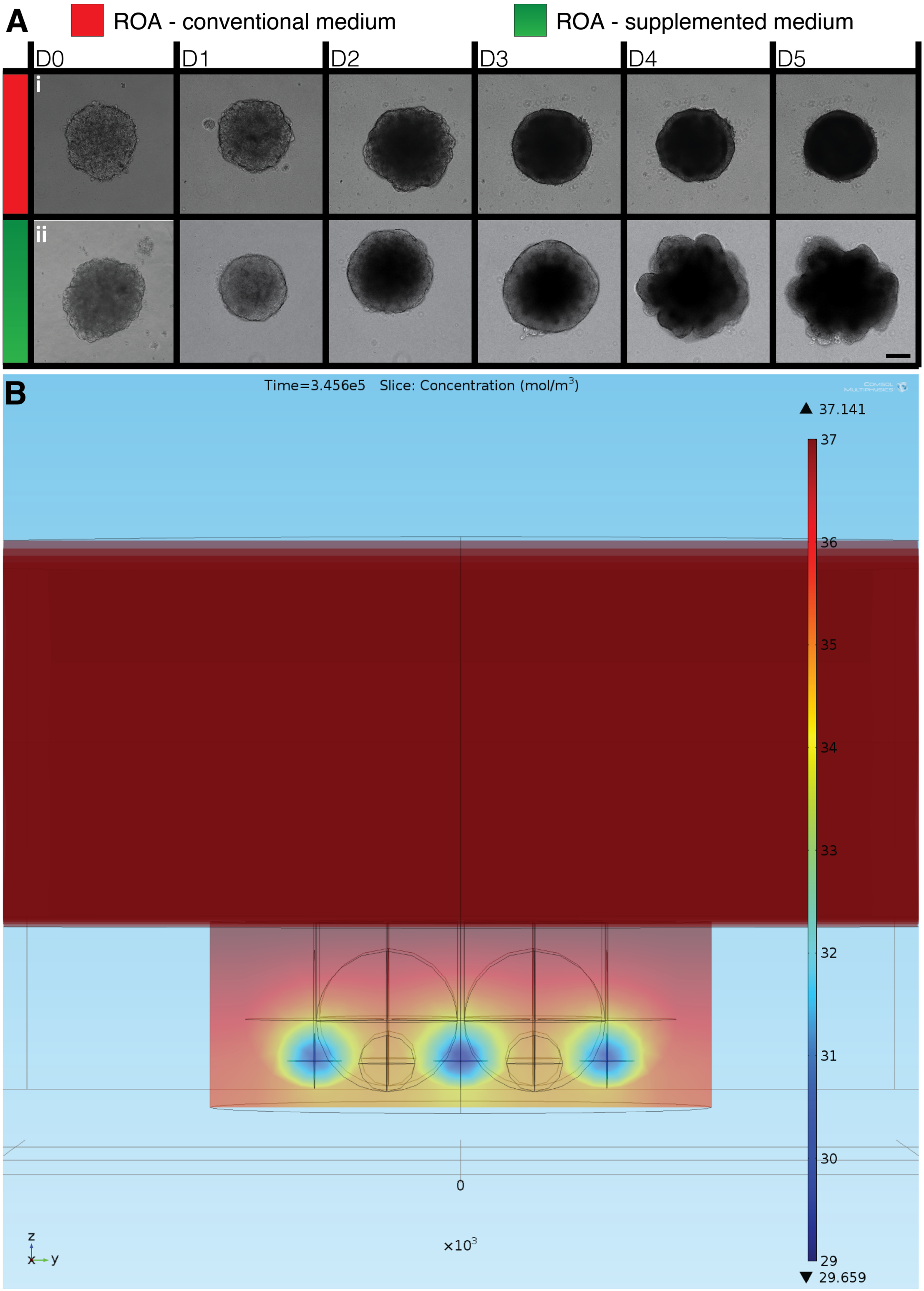

Supplementary Figure 2: A) Representative bright-field images of ROAs development over the first five days of differentiation in two different media composition; i) the conventional medium formulation on milliwell arrays, in red, and ii) the supplemented medium formulation on milliwell arrays, in green. **B)** Representative image of the last time point (day 4) of the diffusion-reaction simulation of seven cellular spheres in one milliwell array with 840 μL of medium.

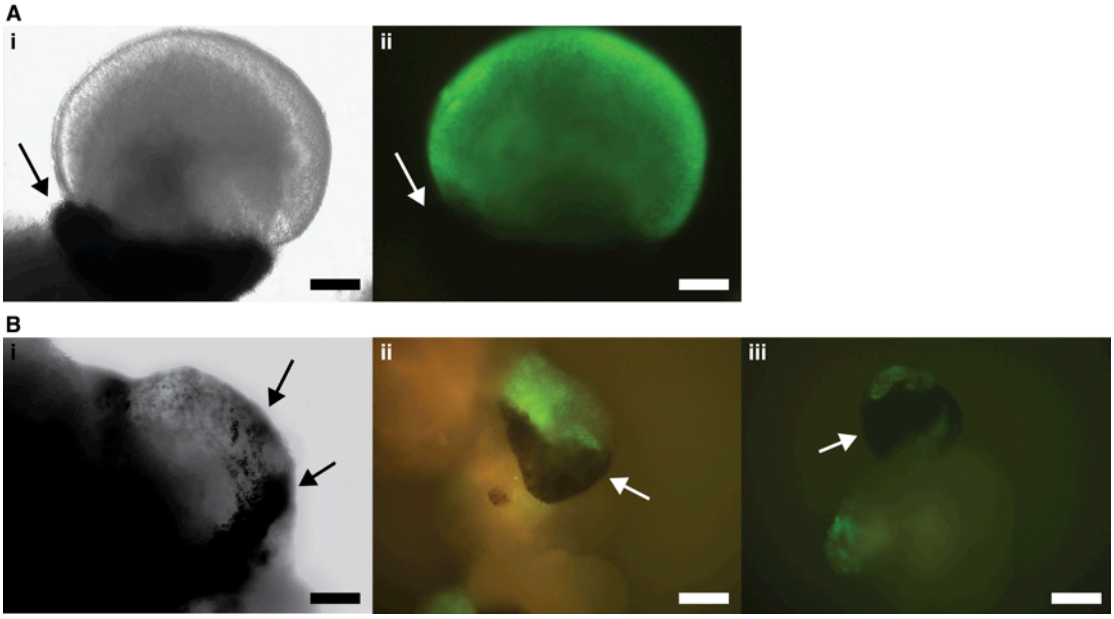

Supplementary Figure 3: A-Biii) Variability of RPE growth on ESC-derived retinal organoids. **(Ai-Bi)** Bright-field and (Aii-Bii-Biii) fluorescent pictures showing different examples of RPE development in retinas at day 19 (Ai-Aii) and day 17 (Bi-Biii) of culture respectively. Photoreceptors in green. RPE in black (arrows). Scale bars: 100 μm in A-Bi and 200 μm in Bii-Biii.

Supplementary Table 1: Primer list for qPCR.

**Supplementary Table S1.:**	**Primer list for qPCR**	
**Gene**	**Forward primer (5' -> 3')**	**Reverse primer (5' -> 3')**
mGnat2	gagggagtcacctgcatcat	aagactcgtgcatgcgatt
mRxry	tcctccaggaatcaacttgg	agctgctgacactgttgacc
mCrx	ccccaatgtggacctgat	ggctcctggtgaatgtggt
mOnecut1	agaccttccggaggatgtg	ttgctctttccgtttgcag
mVsx2	cgtaagaagcggcgacac	tctgggtagtgggcttcatt
mRhodopsin	acctggatcatggcgttg	tgccctcagggatgtacc
mTrb2	gcactgaagaatgagcagac	acacagagctcatctttgtc
mErl8	actggacagttcgtgtact	gcttcactcgagtcttctt
mRax	cgacgttcaccacttaccaa	tcggttctggaaccatacct
mTll1	tgattgcaaggaagccgagt	ggaatggcactgatcaccca
mLhx2	ccaccagcttcggacaatga	accagacctggaggactctc
mSix3	ctgcccaccctcaacttctc	gcaggatcgactcgtgtttgt
mSix6	caaccccagcaaaaagcgag	cctgctgctggagtctgttt
mOtx2	gactgcagggcagagacg	ggtagatttggagtgacggaac
mPax6	gcttggtggtgtctttgtca	tttgcatctgcatgggtct
